# Denaturation and in Vitro Gastric Digestion of Heat-Treated Quinoa Protein Isolates Obtained at Various Extraction pH

**DOI:** 10.1007/s11483-016-9429-4

**Published:** 2016-04-23

**Authors:** Geraldine Avila Ruiz, Mauricio Opazo-Navarrete, Marlon Meurs, Marcel Minor, Guido Sala, Martinus van Boekel, Markus Stieger, Anja E. M. Janssen

**Affiliations:** Food and Biobased Research, Wageningen University and Research Centre, P.O. Boxs 17, 6700 AA Wageningen, Netherlands; Food Quality and Design Group, Wageningen University and Research Centre, P.O. Boxs 17, 6700 AA Wageningen, Netherlands; Food Process Engineering, Wageningen University and Research Centre, P.O. Box 17, 6700 AA Wageningen, Netherlands; Division of Human Nutrition, Wageningen University and Research Centre, P.O. Box 8129, 6700 EV Wageningen, Netherlands

**Keywords:** Quinoa, Protein, Heat processing, Denaturation, Digestibility, Extraction pH

## Abstract

The aim of this study was to determine the influence of heat processing on denaturation and digestibility properties of protein isolates obtained from sweet quinoa (*Chenopodium quinoa* Willd) at various extraction pH values (8, 9, 10 and 11). Pretreatment of suspensions of protein isolates at 60, 90 and 120 °C for 30 min led to protein denaturation and aggregation, which was enhanced at higher treatment temperatures. The in vitro gastric digestibility measured during 6 h was lower for protein extracts pre-treated at 90 and 120 °C compared to 60 °C. The digestibility decreased with increasing extraction pH, which could be ascribed to protein aggregation. Protein digestibility of the quinoa protein isolates was higher compared to wholemeal quinoa flour. We conclude that an interactive effect of processing temperature and extraction pH on in vitro gastric digestibility of quinoa protein isolates obtained at various extraction pH is observed. This gives a first indication of how the nutritional value of quinoa protein could be influenced by heat processing, protein extraction conditions and other grain components.

## Introduction

Quinoa has a balanced amino acid profile with high amounts of lysine and methionine. Sweet varieties of quinoa are more promising to provide high-quality protein in a more economic and sustainable way than the bitter quinoa varieties. More economic because saponins do not have to be removed, which saves in post-harvest processing. More sustainable because sweet varieties have been successfully adapted to North West European climates and soils, and could also be adapted to other regions in the world, making local quinoa production possible [1, 2].

Protein functionality is an important aspect to evaluate the potential of a new protein and give guidance for usage in applications. To avoid influences from other grain components in assessing the protein potential as a food ingredient, the protein can best be isolated from the grain for subsequent analysis. Conventionally, solvent extraction is used to isolate protein from plant material. During this process, protein properties and thus functionality can be affected [3]. Only a few studies have examined the impact of extraction conditions on functional properties of quinoa protein so far, and only our previous study has investigated properties of quinoa protein from sweet quinoa (saponin-free) [4–6]. The absence of saponins has been found to influence protein efficiency ratio, nitrogen solubility, emulsifying and foaming properties [3]. Next to extraction conditions, post-extraction processing can also influence protein properties. A few recent studies have investigated the effects of post-extraction heating on some properties of Quinoa Protein Isolates (QPI). We previously found that QPI suspensions started to gel at about 70 °C when extracted at pH 8 and 9 but no gelation was observed when extracted at pH 10 or 11. Maekinen et al. (2015) reported that cold-set QPI gels were finer, more regularly structured and had a higher storage modulus when QPI suspensions were heat-treated (100 °C, 15 min) at pH 10.5 than when heat-treated at pH 8.5 [7]. Silva et al. (2015) found that heat treatments (100 °C, 30 min) of quinoa protein fractions containing anti-nutritional factors increased in vitro protein digestibility. To the best of our knowledge, no studies have investigated the effect of varying heat processing parameters on protein denaturation and digestibility of QPIs. Protein denaturation and digestibility are main determinants of protein quality and would be important for application of quinoa (protein) in food products [8]. Gastric protein digestibility is a first indicator of overall protein digestibility and nutritional value of the protein [9, 10] [11–13]. Therefore, in the present study, we examined how heat processing at different temperatures influenced denaturation properties and in vitro gastric digestibility of sweet quinoa protein isolated at various extraction pH values. Based on literature, we hypothesize that heat processing in the temperature range of 60 to 120 °C increases in vitro gastric digestibility of the quinoa protein at mildly alkaline extraction pH and decreases the digestibility at strongly alkaline extraction pH.

## Material and Methods

### Materials

Quinoa seeds (*Chenopodium quinoa* Willd) of the sweet variety *Atlas* were supplied by the Agricultural Research Institute (INIA) in Santiago, Chile. Petroleum ether (boiling range 40–60 °C) was used (Sigma-Aldrich Chemie GmbH, Schnelldorf, Germany). Chemicals for preparation of the simulated gastric juice were purchased from Sigma-Aldrich, Inc. (St. Louis, MO, U.S.A.).

### Preparation of Quinoa Protein Isolates

Quinoa seeds were ground with a Fritsch Mill Pulverisette 14 (Idar-Oberstein, Germany) using a speed of 7000 rpm and sieved through a 200 μm sieve. The flour was defatted in a Soxhlet using petroleum ether with a sample-to-solvent mass ratio of 1:5 for 24 h [14]. The petroleum ether was removed by evaporation. The defatted flour was suspended in deionized water (10 % *w*/w) and the pH was adjusted to 8, 9, 10 and 11 by addition of 1 N NaOH. The suspensions were stirred for 1 h at room temperature and centrifuged for 20 min at 6000 g and 10 °C. The obtained supernatants were acidified to pH 5.5 by addition of 1 N HCl. The suspensions were centrifuged for 30 min at 13,000 g and 10 °C. The precipitated pellets were re-suspended in deionized water (5 % *w*/w). To rinse remaining salts the suspensions were centrifuged for 20 min at 11,000 g and 10 °C, re-suspended in deionized water (5 % *w*/w) and neutralized by addition of 1 N NaOH. The suspensions were frozen by dipping into liquid nitrogen and subsequently freeze-dried for 72 h (Chris Epsilon 2-6D Freeze Dryer, Osterode am Harz, Germany). The dried protein isolates were ground with a spoon for about 30 s to obtain powders. Isolates were obtained in duplicate from two separate extractions.

### Determination of Protein Yield and Purity

8 to 15 mg QPI was weighed in tin cups and dried overnight at 60 °C. The nitrogen content was determined by sample combustion in a Dumas Flash EA 1112, Series NC analyzer (Wigan, UK) and converted to crude percentage of protein using a protein factor of 5.85 [4, 15, 16]. Measurements were performed in duplicate. Protein yield and protein purity were calculated as follows:

$$ \mathrm{Protein}\ \mathrm{yield}\left(\%\right)=\frac{\mathrm{protein}\ \mathrm{content}\ \mathrm{isolate}\ \left(\%\right)\times \mathrm{dry}\kern0.5em \mathrm{isolate}\ \left(\mathrm{g}\right)}{\mathrm{protein}\ \mathrm{content}\ \mathrm{flour}\ \left(\%\right)\times \mathrm{flour}\ \left(\mathrm{g}\right)}\times 100 $$

$$ \mathrm{Protein}\ \mathrm{purity}\left(\%\right)=\frac{\mathrm{protein}\ \mathrm{content}\ \mathrm{isolate}\ \left(\%\right)\times \mathrm{dry}\ \mathrm{isolate}\ \left(\mathrm{g}\right)}{\mathrm{dry}\ \mathrm{isolate}\kern0.5em \left(\mathrm{g}\right)}\times 100 $$

### Heat Processing of Quinoa Protein Isolates

Suspensions of the QPIs obtained at the different extraction pH values were prepared at protein concentrations 1, 5 and 20 % *w*/w in deionized water and stirred for 1 h at room temperature. For the heat processed samples, the suspensions were heat-treated in an Eppendorf thermomixer (Eppendorf AG, Hamburg, Germany) for 30 min at 60, 90 and 120 °C and then cooled down to room temperature. The temperatures were selected based on temperatures used in applications and to test within a wide range of temperatures. A temperature of 90 °C represents pasteurization conditions, while a temperature of 120 °C is representative for sterilization conditions. Treatment at 60 °C was chosen as mild heating temperature without causing denaturation of the quinoa protein. The terms “processing temperature of 20 °C″ and “unprocessed” refer to the incubation of QPI suspensions at 20 °C without further treatment.

### Determination of Molecular Weight Distribution

Sodium dodecyl sulphate–polyacrylamide gel electrophoresis (SDS–PAGE) was used to determine the molecular weight distribution of the quinoa protein isolate fractions. Heat-processed and unprocessed suspensions of 1 % *w*/w protein concentration were prepared. The suspensions were then re-suspended in deionized water (pH 6.5 ± 0.1) and centrifuged for 1 min at 13,000 g to obtain the solubilized protein. The supernatants were diluted with 1 x NuPAGE® LDS Sample Buffer and deionized water before applying the samples to the gel. NuPAGE® Novex® Bis-Tris Gels (1–200 kDa) containing 12 % acrylamide (4 % acrylamide stacking gel) were used. The molecular weight markers were from NuPAGE® Novex® (Mark 12™ Unstained Standard, 2.5–200 kDa). Protein bands were stained with Simply Blue™ SafeStain.

### Determination of Thermal Properties

The thermal properties of the QPIs were assessed by Differential Scanning Calorimetry (DSC). Heat-processed and unprocessed suspensions of 20 % *w*/w protein concentration were prepared. Hermetically sealed aluminum pans were filled with 25–50 mg of heat-processed or unprocessed QPI suspensions. DSC samples were heated at a rate of 10 °C/min from 20 to 140 °C using a PerkinElmer Diamond series differential scanning calorimeter equipped with an intracooler 2P. A double, empty pan was used as reference. The denaturation parameters were calculated using Pyris Software (Version 11, PerkinElmer) with the denaturation temperature (T_d_) value corresponding to the maximum transition peak and the transition enthalpy (denaturation enthalpy ΔH) calculated from the area below the transition peaks. Measurements were performed in duplicate for isolates obtained in duplicate.

### Determination of in Vitro Gastric Protein Digestibility

Simulated gastric juice was prepared according to [17, 18]. Pepsin (1 g L-1), mucin (1.5 g L-1), and NaCl (8.775 g L-1) were dissolved in Milli-Q water and the pH was adjusted to 2.0 with 2 M HCl. Heat-processed and unprocessed QPI suspensions, as well as suspensions of whole meal quinoa flour (5 % *w*/w protein, 2 mL), were prepared and added to 50 mL of simulated gastric juice in a jacketed glass vessel connected to a water bath at 37 °C (Julabo GmbH, Seelbach, Germany). The vessel was sealed with Parafilm (Pechiney Plastic Packaging, Inc., IL, U.S.A.) to avoid evaporation and the gastric juice solutions were stirred at 100 rpm. Samples of 1 mL were taken after 0, 5, 10, 15, 20, 30, 45, 60, 90, 120, 150, 180, 240 and 360 min and heated under stirring in a pre-heated Eppendorf thermomixer (Eppendorf AG, Hamburg, Germany) at 90 °C and 1400 rpm for 5 min to inactivate pepsin [19]. All measurements were performed in triplicate.

### Determination of Degree of Hydrolysis (DH)

The degree of hydrolysis (DH) is defined as the percentage of cleaved peptide bonds over the total number of peptide bonds. The latter was calculated as follows:

$$ \mathrm{Total}\ \mathrm{number}\ \mathrm{of}\ \mathrm{peptide}\ \mathrm{bonds}=\frac{\mathrm{average}\ \mathrm{molecular}\ \mathrm{weight}\ \mathrm{of}\ \mathrm{amino}\ \mathrm{acids}\ \left(\mathrm{kDa}\right)}{1000\ \mathrm{g}\ \mathrm{protein}\ } $$

To estimate DH, the OPA method was used as described by Luo et al. (2015). The OPA reagent was prepared and stored in a bottle covered with aluminum foil to protect the reagent from light. A spectrophotometer DU 720 (Beckman Coulter Inc. Pasadena, CA, U.S.A) was set at 340 nm with 1.5 mL OPA reagent +0.2 mL Milli-Q water. Serine standard solutions of 200 μL of 50 mg/L, 100 mg/L, 150 mg/L and 200 mg/L were added to 1.5 mL OPA reagent and mixed. The solutions were measured with the spectrophotometer after standing for 3 min. The samples were pipetted into the Amicon Ultra-0.5 10 K Centrifugal Filter Units (Millipore, USA) and centrifuged for 20 min at 14,000 g. All measurements were performed in triplicate.

### Size Exclusion Chromatography (SEC)

The peptide profile after digestion was analyzed using SEC Ultimate 3000 UHPLC system (Thermo Scientific, MA, U.S.A.) equipped with a TSKgel G2000SWxl column (Tosoh Bioscience LLC, PA, U.S.A.). 0.1 mL sample was used for analysis. The running buffer consisted of acetonitrile and 70 % Milli-Q water with 0.1 % Trifluoro Acetic Acid (TFA). The flow rate of the running buffer was 1 mL/min and the UV detector was set at 214 nm. In order to standardize the molecular weight range of the chromatographic separation, the following purified proteins and amino acids were used for calibration: carbonic anhydrase (29 kDa), α-lactalbumin (14.1 kDa), aprotinin (6.51 kDa), insulin (5.7 kDa), bacitracin (1.42 kDa) and phenylalanine (165 Da) (Sigma-Aldrich, Inc., St. Louis, MO, U.S.A.). The area under the curves was determined and the relative area for each segment calculated. All measurements were done in triplicate.

## Results and Discussion

### Protein Yield and Purity

**Fig. 1:**
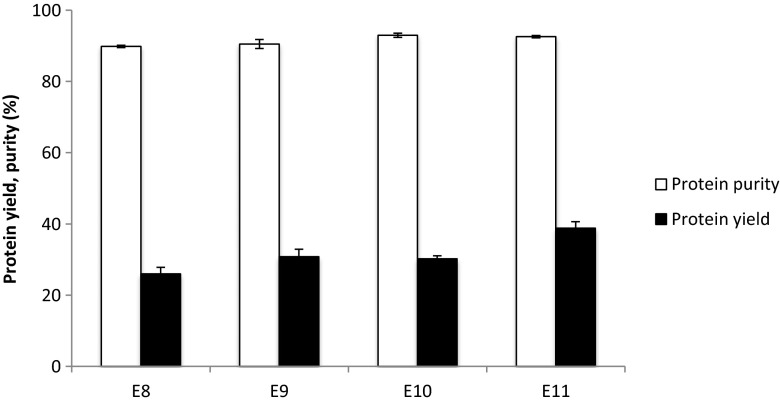
Protein yield and protein purity on dry matter basis of the quinoa protein isolates E8, E9, E10 and E11. Error bars represent the standard deviation based on duplicate extraction experiments

When extracting quinoa protein in a pH range of 8–11, a protein purity of 90–93 % was obtained (Fig. 1). These values are the highest reported in literature so far [4, 6, 20–23]. In our previous study, we used a similar extraction protocol, only the alkalinization time was longer and the precipitation pH lower, resulting in a lower protein purity (82–88 %) [20]. Protein yield increased from 24 to 37 % when increasing the extraction pH from 8 to 11. These values are lower than in our previous study (35–50 % going from extraction pH 8 to 11) but they also increased with extraction pH. For industrial production of quinoa protein isolates, this means that the extraction pH would need to be controlled carefully.

### Thermal Properties

Unprocessed and processed 20 % QPI suspensions showed an endotherm from 96 to 102 °C (denaturation temperature range) (Figs. 8, 9, 10, 11, 12), which is in line with denaturation temperatures (T_d_) previously found for quinoa, amaranth and sunflower protein. These denaturation temperatures have been attributed to 11S globulin [4, 16, 20, 24, 25]. Therefore, we assume that the endotherm found in our study also mainly corresponds to 11S globulin. There was no significant change in T_d_ with processing temperature, but T_d_ decreased with increasing extraction pH. This decrease was also observed by Martínez & Añón (1996) for amaranth protein and indicates that protein is less heat-stable when extracted at higher pH [24].

**Fig. 2:**
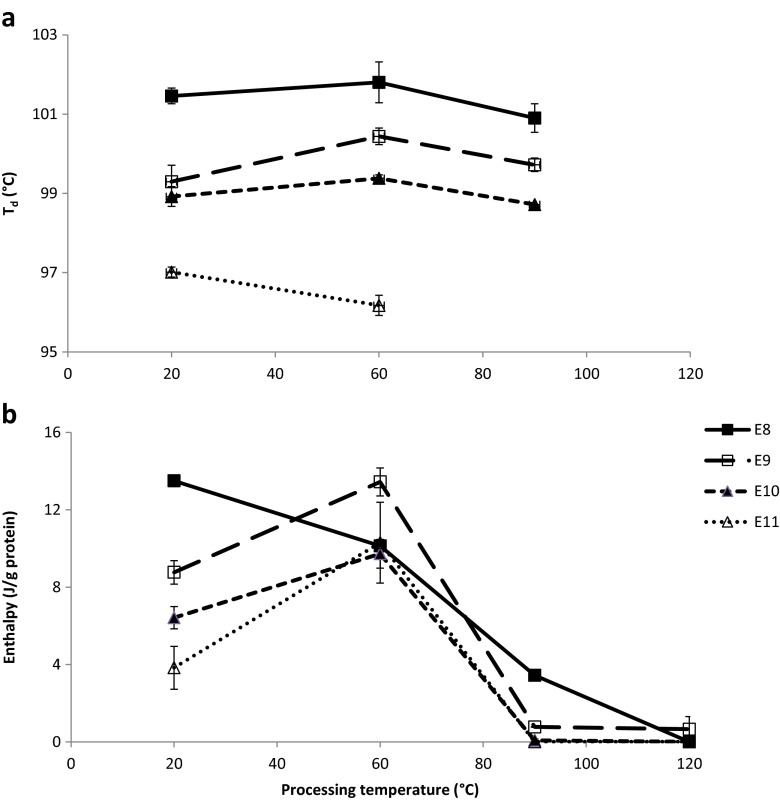
**a** Denaturation temperature (T_d_) and **b** denaturation enthalpy (ΔH) of 20 % *w*/w suspensions of QPI E8, E9, E10 and E11 after processing at different temperatures. Data were obtained from DSC measurements

The denaturation enthalpy of the unprocessed QPI suspensions decreased considerably from 13.5 to 3.8 J/g protein with increasing extraction pH (Fig. 2). This trend has also been observed in several other studies on quinoa, amaranth and sunflower protein, showing that the protein is more denatured at higher extraction pH [4, 16, 20, 24, 25]. When QPI suspensions were processed at 90 and 120 °C, the denaturation enthalpy was reduced to 0–3.4 J/g protein. However, the enthalpy was significantly higher after processing at 60 °C than at 20 °C for E9, E10 and E11.

Martínez & Añón (1996) have summarized the notion of denaturation enthalpy to be the result of endothermal processes, e.g. disruption of hydrogen bonds, and exothermal processes, e.g. protein aggregation and disruption of hydrophobic interactions. The higher denaturation enthalpy (or transition enthalpy) of E9, E10 and E11 at 60 °C might thus indicate a conformation of the protein that was stabilized by a greater extent of hydrophobic interactions and/or hydrogen bonds and that cost more transition energy than at 20, 90 or 120 °C. The exception was E8, which showed a continuous decrease in enthalpy from 20 to 120 °C. Based on the notion of denaturation enthalpy of Martínez & Añón (1996) it might be that at an extraction pH of 8 the protein initially contained a higher degree of hydrophobic interactions and/or hydrogen bonds as compared to the protein obtained at other extraction pH values. These molecular interactions might have decreased in number from a processing temperature of 20 to 60 °C in contrast to the other extraction pH values, where the protein initially had undergone more extensive conformational changes due stronger alkaline extraction conditions, resulting in a different degree of molecular interactions after processing at 60 °C. In summary, the effect of processing temperature on the thermal properties of QPIs seemed to depend on the protein properties predetermined by the extraction pH.

### Protein Fractions

**Fig. 3:**
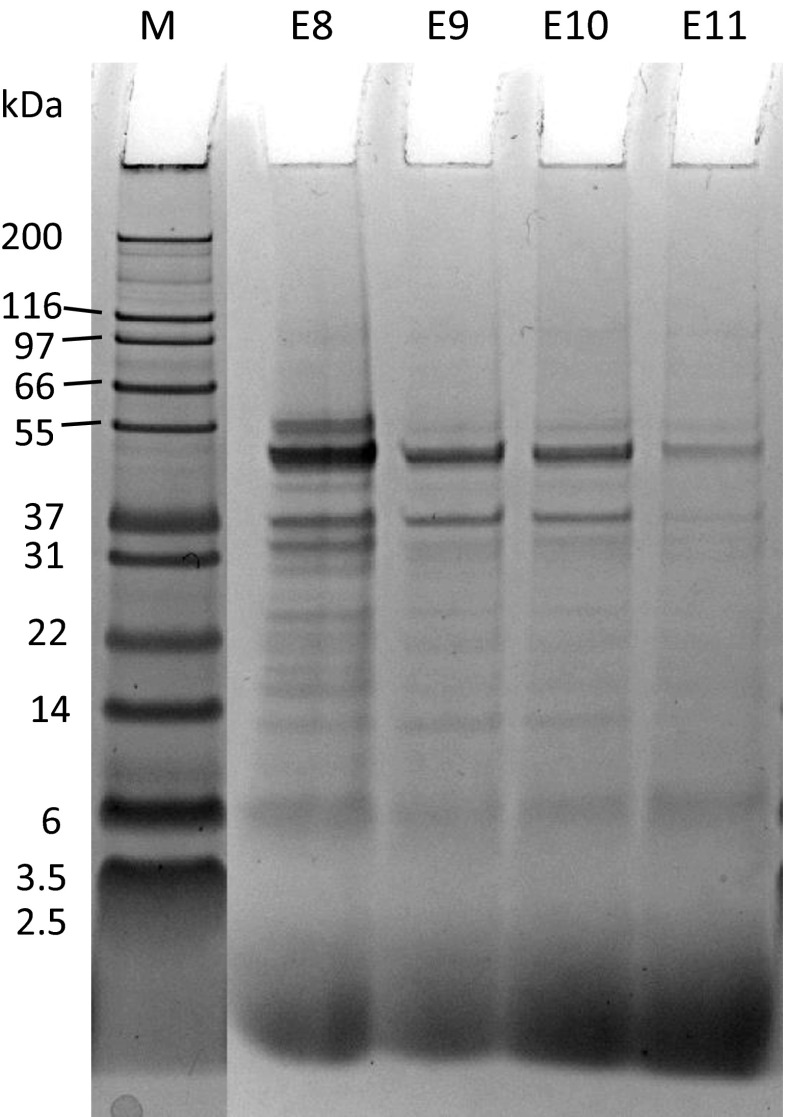
SDS-PAGE profile of the unprocessed QPIs E8, E9, E10 and E11. Lane M: molecular weight marker

SDS profiles showed major bands at 50 kDa for all QPIs and at 37 kDa for E8, E9 and E10 (Fig. 3). The bands of E8 were the most intense and decreased in intensity with increasing extraction pH. The SDS profiles were similar to the ones of previous quinoa protein studies, suggesting a correspondence of the bands at 50 kDa to 11S globulin [4, 20, 26]. Furthermore, bands at 37 kDa might correspond to the acidic subunit and bands at 23 kDa might be attributed to the basic subunit of 11S globulin. Alkali is known to cause disulfide bond cleavage, resulting in the dissociation of 11S globulin into acidic and basic subunits of 32–39 kDa and 22–23 kDa, respectively [27].

**Fig. 4:**
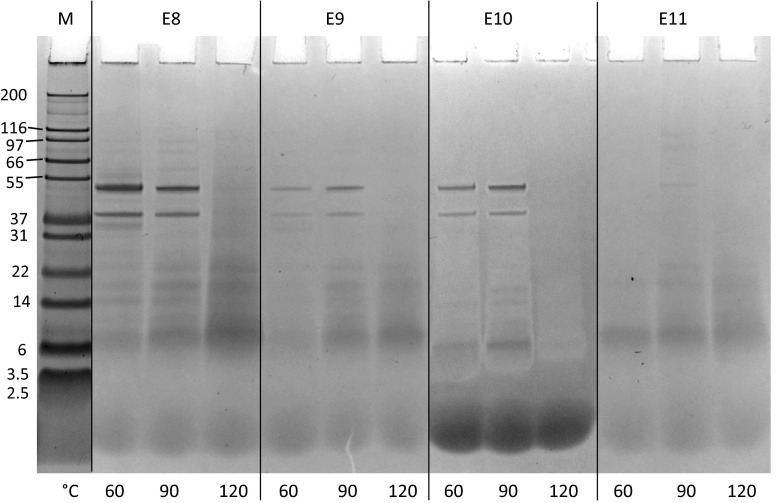
SDS-PAGE profile of the QPIs E8, E9, E10 and E11 heat-treated for 30 min at 60, 90 and 120 °C. Lane M: molecular weight marker. The gel of E10 seems to be overloaded at the bottom. E10 was run on a different gel and is shown in Fig. 13

After heat processing, the SDS profiles showed less bands with less intensity for all QPIs (Fig. 4). In some lanes specific bands were even not visible anymore.

The disappearance of bands with increasing processing temperature indicates enhanced protein aggregation to protein particles larger than 200 kDa or to insoluble protein particles that remained in the pellet after centrifuging the heat-processed protein suspensions. Protein aggregation might have resulted from increased protein dissociation and subunit interactions and re-association to larger (insoluble) aggregates as reported for heat-processed soy protein (0–30 min at 80 and 100 °C) [28, 29]. DSC results showed higher denaturation enthalpies of the unprocessed and 60 °C-processed QPI suspensions compared to the suspensions processed at 90 and 120 °C. As described before, the higher enthalpies might result from more hydrophobic interactions, hydrogen bonds but also from increased protein aggregation, according to Martínez & Añón (1996). Based on the results of SDS and DSC, it seems likely that protein aggregation leads to insoluble particles remaining in the pellet, especially at 120 °C (less protein on the SDS gels), while the aggregates seem to be less capable to undergo a heat-induced phase transition up to a temperature of 140 °C (maximum temperature reached during DSC measurements) compared to protein treated at 60 °C.

### In Vitro Gastric Protein Digestibility of Quinoa Protein Isolates

**Fig. 5:**
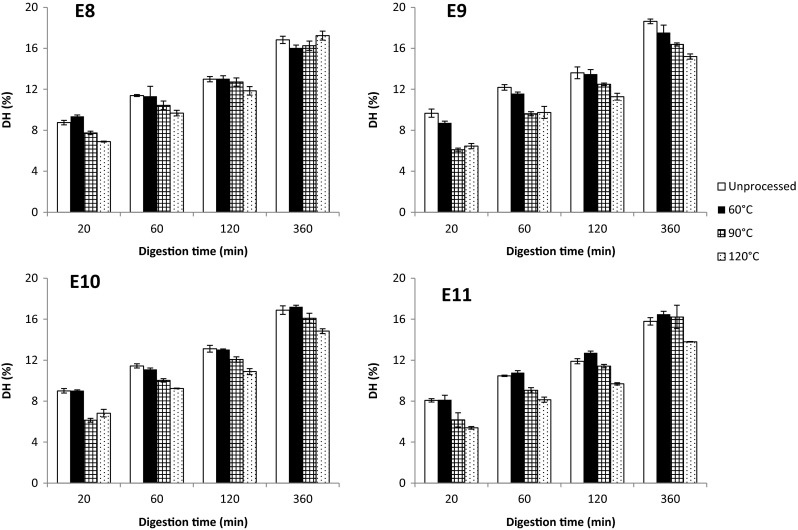
Degree of hydrolysis (DH) of 5 % *w*/w suspensions of QPI E8, E9, E10 and E11 processed at different temperatures and subsequently digested for different time periods

Gastric digestibility of the QPIs was studied in vitro simulating physiological conditions and was indicated as the degree of protein hydrolysis (% peptide bonds cleaved by pepsin of total bonds). The degree of hydrolysis (DH) of unprocessed and processed 5 % QPI suspensions sharply increased within the first 20 min and further increased at a slower rate in the following hours (Fig. 5). The hydrolysis profiles compare to those of whey protein and egg white protein obtained by Luo et al. (2015) at the same protein concentration, and under the same digestion and measurement conditions. When interpolating the DH values of the QPI suspensions treated at 90 °C to a digestion time of 3 h, the DH of quinoa protein was slightly lower (13–14 %) than the DH of whey protein (15 %) but higher than the DH of egg white protein (11 %), both pre-treated for 30 min at 90 °C and digested for 3 h.

**Fig. 6:**
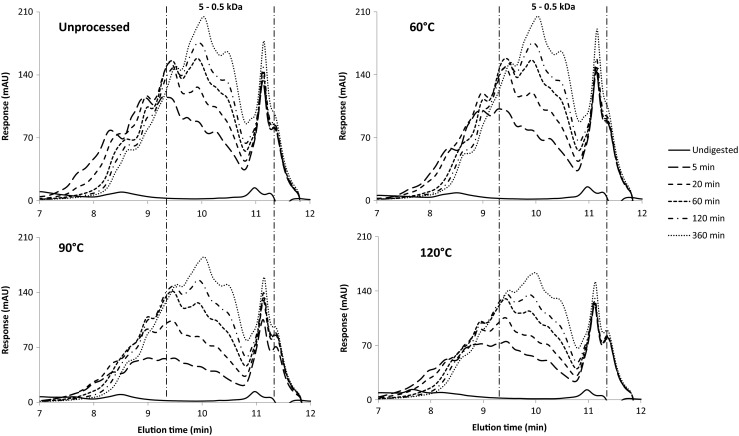
HPLC chromatograms of 5 % *w*/w suspensions of QPI E9 processed at different temperatures and subsequently digested for different time periods. Size exclusion chromatography is used for separation. This means that larger peptides have a low elution time. See Fig. 14, 15, 16 for the HPLC chromatograms of E8, E10 and E11

HPLC chromatograms showed that when digesting unprocessed and processed QPI suspensions for 5–360 min higher amounts of peptides ranging from 0.5 to 5 kDa were obtained (Figs. 6, 14, 15, 16). The peaks in the molecular size range of 0.5–5 kDa became larger and moved to a smaller size range with increasing in vitro digestion time. As digestion progressed, pepsin cleaved increasingly more peptide bonds, resulting in smaller molecules. When comparing processing temperatures, the chromatograms did not significantly change from 20 to 60 °C. However, at 90 and 120 °C, the response areas were significantly smaller compared to 20 and 60 °C. This is most clearly visible after 5 and 20 min of digestion. This finding could be confirmed by DH measurements (Fig. 5): the DH was reduced overall at 90 and 120 °C compared to 20 and 60 °C. Similar observations were made for lupine protein [30]. A heat treatment at 60 °C for 30 min did not change the digestibility of lupine protein compared to the untreated sample, while a heat treatment at 90 °C for 30 min did reduce the digestibility. The reduction in the DH at higher processing temperature was enhanced at higher extraction pH.

These results suggest that pepsin was less effective after heat-treatment of the QPI suspensions. This might be explained by the heat-induced change in protein conformation, molecular interactions and protein aggregation as indicated by DSC and SDS results. Increased protein aggregation after the heat treatments might have reduced the accessibility of pepsin. Impairment of protein digestibility for pepsin has already been previously correlated with stronger protein crosslinking when cooking sorghum [31]. The in vitro digestibility of sorghum protein using pepsin has therefore been validated as an indicator for the degree of protein crosslinking. This relation might also be valid for quinoa protein.

If this is the case, the fact that the reduction in the DH at higher processing temperature was enhanced at higher extraction pH can be explained with increased protein crosslinking. This might also be deduced from SDS results: with an increasing extraction pH and processing temperature, the degree of protein aggregation, possibly as a result of protein crosslinking, seemed to be higher. However, DSC results implied that the protein suspensions from a high extraction pH (10 and 11) and processing temperature (90 and 120 °C) were only slightly capable or not capable at all to undergo a heat-induced phase transition. Therefore, not a greater extent of protein aggregation or crosslinking seemed to be impairing enzyme action more under these harsher conditions, but a more heat-resistant type of protein aggregation or crosslinking.

The extraction pH had almost no influence on the DH when comparing pH values of the unprocessed suspensions and of the processed suspensions at 60 and 90 °C (Fig. 5). This means that the effects of extraction pH observed on the physical properties of unprocessed QPIs and processed QPIs at 60 and 90 °C were not clearly transferred to in vitro gastric digestibility. At 120 °C, the rate of DH was only slightly reduced at extraction pH 11 compared to the other extraction pH values. These results show a bigger impact of processing temperature on the DH of quinoa protein compared to extraction pH.

We conclude that heat treatment for 30 min at 90 and 120 °C impairs in vitro gastric digestibility of protein in QPIs.

### Gastric in Vitro Protein Digestibility of Whole Quinoa Flour

**Fig. 7:**
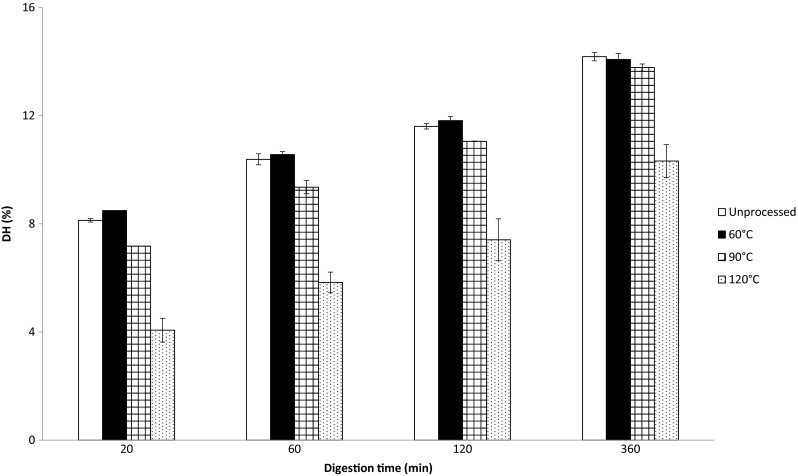
Degree of hydrolysis (DH) of wholemeal quinoa flour (5 % *w*/w protein) processed at different temperatures and subsequently digested for different time periods

**Fig. 8:**
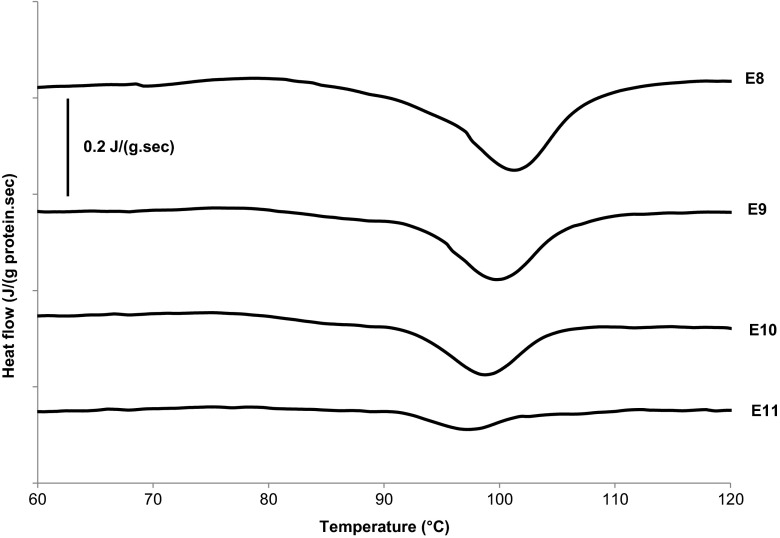
DSC thermograms of untreated 20 % *w*/w suspensions of QPI E8, E9, E10 and E11

**Fig. 9:**
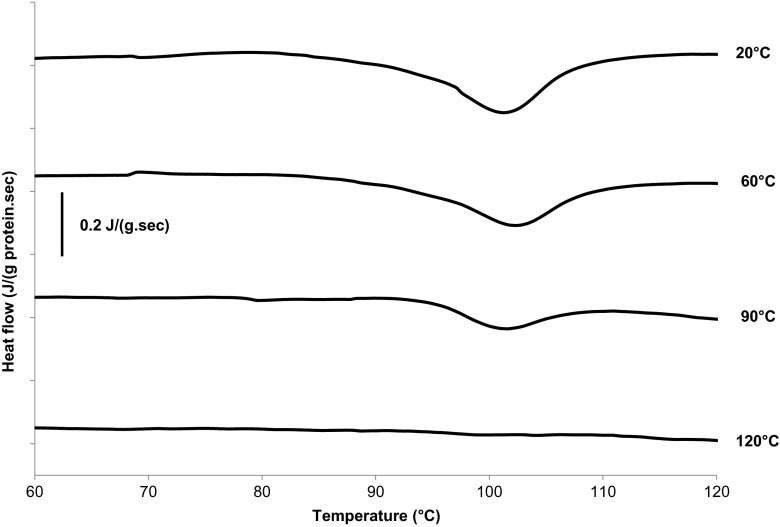
DSC thermograms of 20 % *w*/w suspensions of QPI E8 after processing at different temperatures

**Fig. 10:**
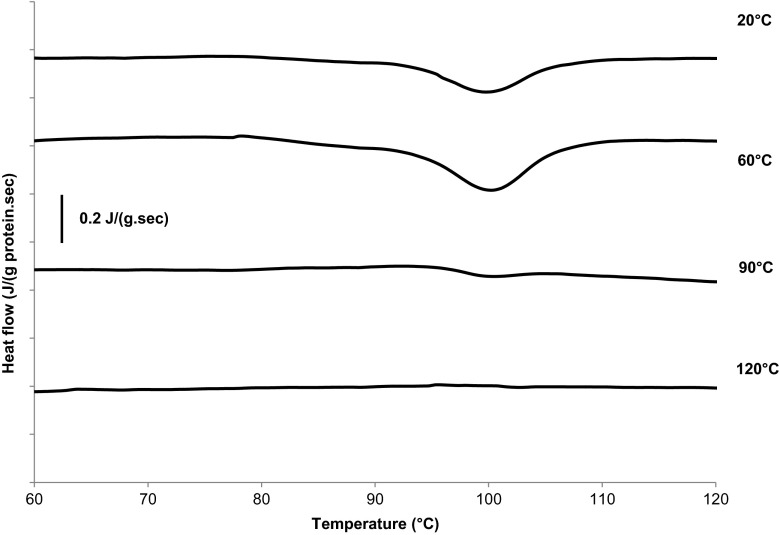
DSC thermograms of 20 % *w*/w suspensions of QPI E9 after processing at different temperatures

**Fig. 11:**
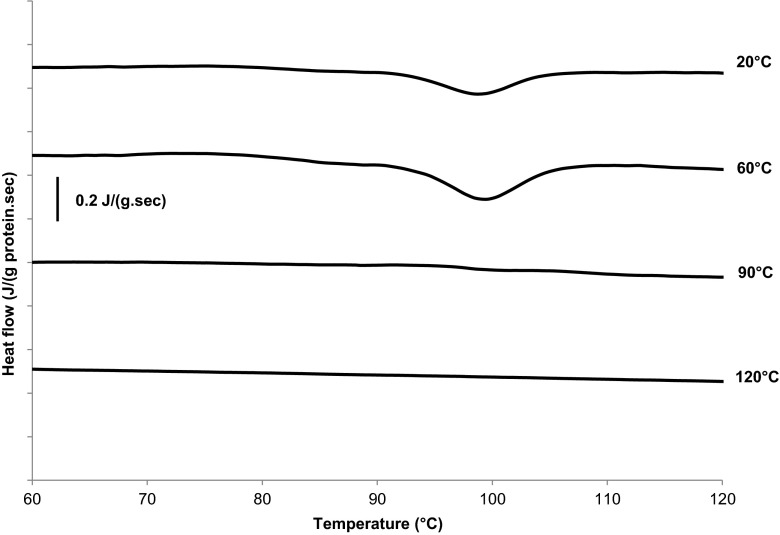
DSC thermograms of 20 % *w*/w suspensions of QPI E10 after processing at different temperatures

**Fig. 12:**
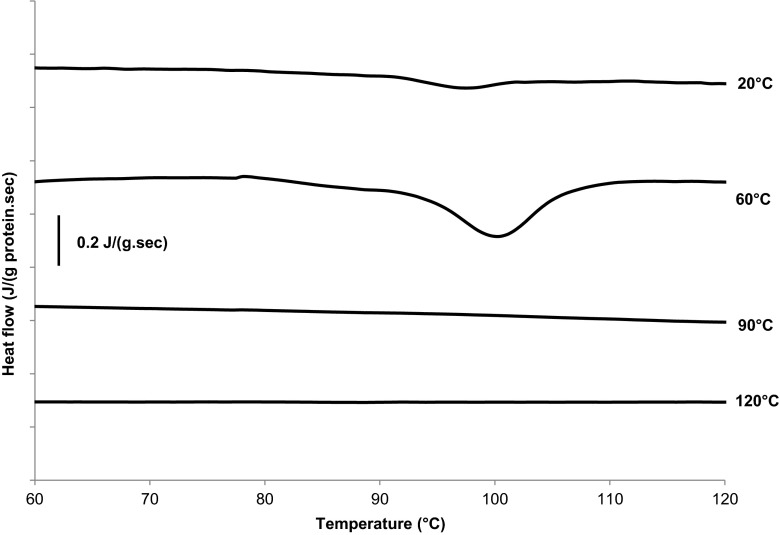
DSC thermograms of 20 % *w*/w suspensions of QPI E11 after processing at different temperatures

**Fig. 14:**
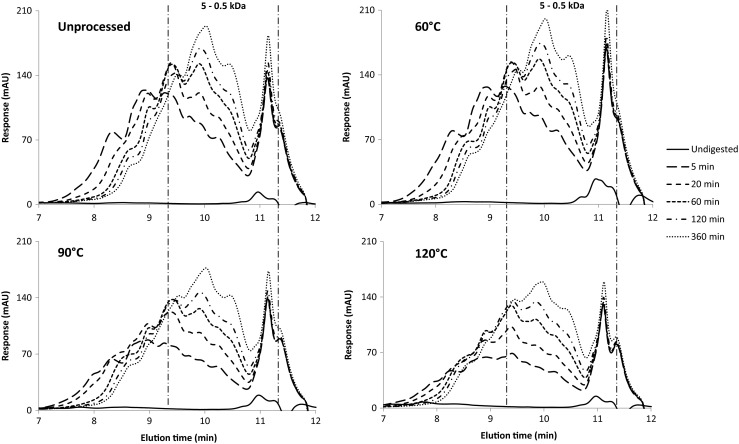
HPLC chromatograms of 5 % *w*/w suspensions of QPI E8 processed at different temperatures and subsequently digested for different time periods. Size exclusion chromatography is used for separation. This means that larger peptides have a low elution time

**Fig. 15:**
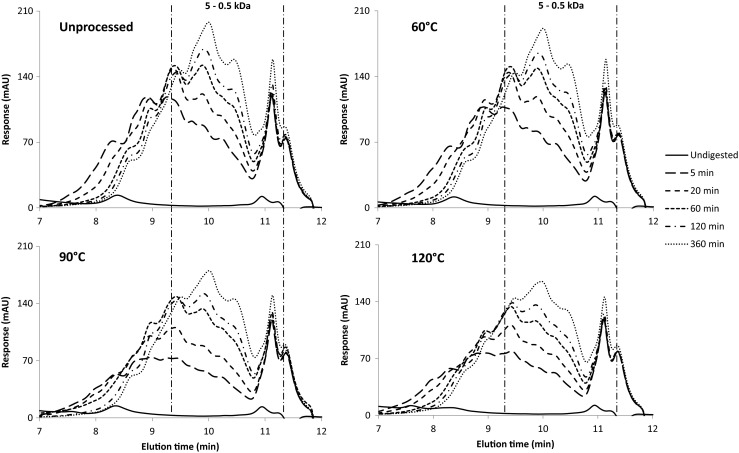
HPLC chromatograms of 5 % *w*/w suspensions of QPI E10 processed at different temperatures and subsequently digested for different time periods. Size exclusion chromatography is used for separation. This means that larger peptides have a low elution time

**Fig. 16:**
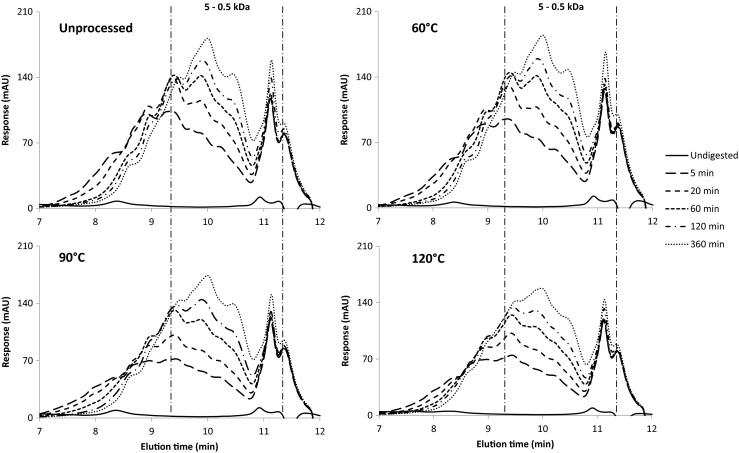
HPLC chromatograms of 5 % *w*/w suspensions of QPI E11 processed at different temperatures and subsequently digested for different time periods. Size exclusion chromatography is used for separation. This means that larger peptides have a low elution time

To examine how protein digestibility in QPIs compares to that in whole quinoa flour, we performed the digestibility study with wholemeal quinoa flour at the same protein concentration. The DH values also increased in time and looked similar to that of the QPIs. However, the DH values were overall lower, especially at 120 °C (Fig. 7). This reduction in DH might be due to the other components present (in higher amounts) in the quinoa flour (mainly starch, fiber and fat). The mere presence of much higher amounts of starch and fiber in the quinoa flour compared to the QPIs might be the responsible factor, but also the behavior of these components at the different processing temperatures might have had an impact on digestibility [32]. The gelatinization of quinoa starch starts from 45 to 54 °C, peaks from 51 to 62 °C and concludes from 64 to 71 °C [33]. At processing temperatures of 60 and 90 °C, there was no large difference in the decrease in DH compared to the protein isolates, indicating that gelatinization did not affect protein digestibility significantly. There was a larger drop in DH from 90 to 120 °C for the quinoa flour compared to the protein isolates. As starch gelatinization did not seem to have an impact on digestibility at lower temperatures, it is possible that at higher temperatures the gelatinized starch interacted with denatured protein (T_d_ = 96–102 °C), thereby hindering enzyme action. Another explanation might be that in contrast to the protein in the flour, the protein in the protein isolates underwent conformational changes during the extraction, which limited the effect of processing temperature on protein digestibility.

## Conclusions

Using the extraction protocol from the present study, we could achieve a very high protein purity, but at the expense of a low protein yield. The degree of denaturation and molecular weight profiles of the QPIs were strongly affected by processing temperature and extraction pH, individually and combined. For QPI’s, extraction pH and processing temperature showed an interactive effect on in vitro gastric digestibility of the protein. Extracting protein from quinoa flour results in a higher protein digestibility when compared to keeping the protein in the flour. For applications, the present findings mean that extraction and processing conditions need to be controlled to optimize protein digestibility. Future research could investigate other functional properties of quinoa protein but also examine ileal and in vivo protein digestibility under various conditions to verify the present findings in more real-life digestion conditions.

## Figures and Tables

**Fig. 13:**
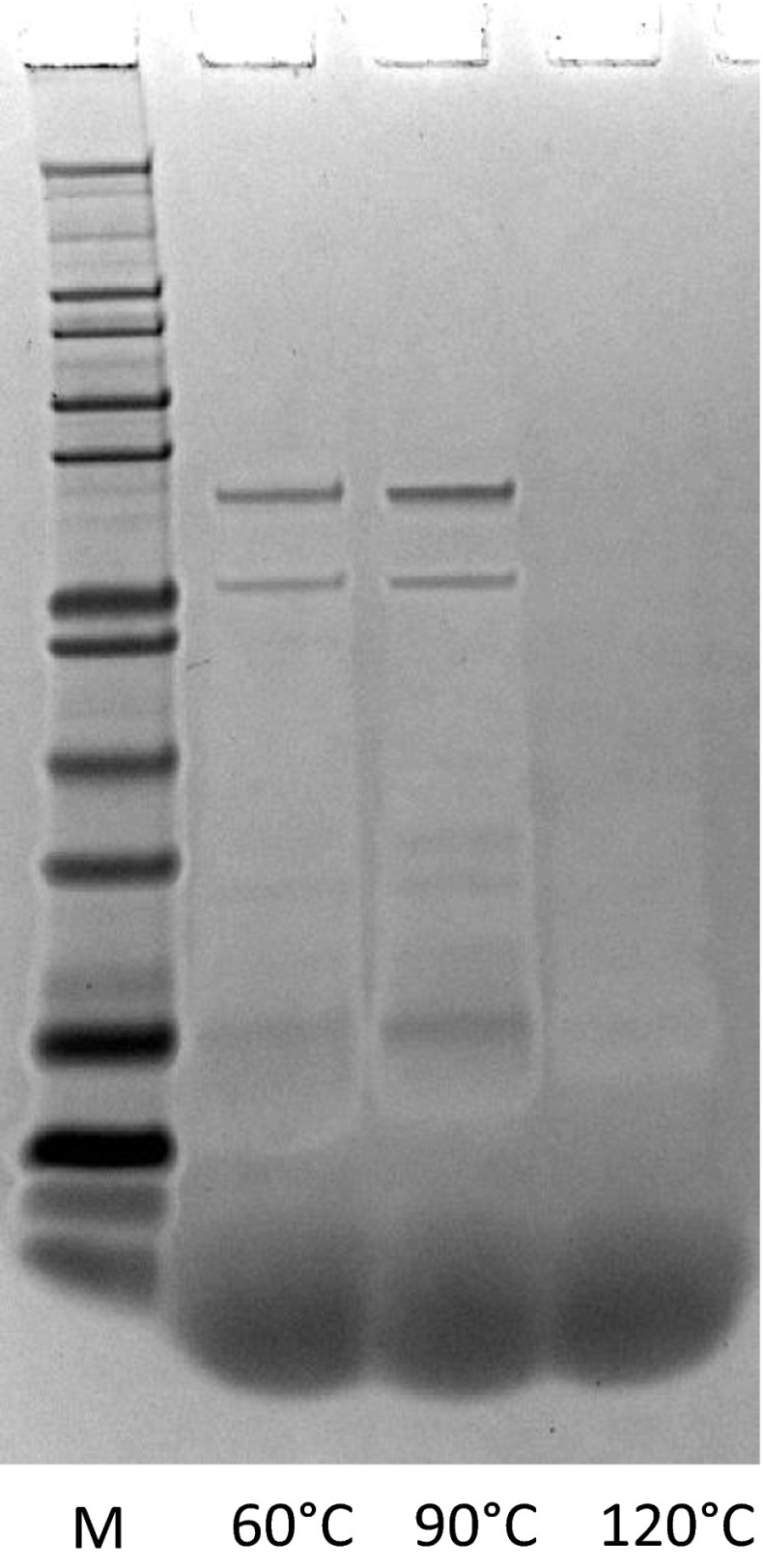
SDS-PAGE profile of the QPIs E10 heat-treated for 30 min at 60, 90 and 120 °C. Lane M: molecular weight marker

## Abbreviations

E8: protein isolated at pH 8
E9: protein isolated at pH 9
E10: protein isolated at pH 10
E11: protein isolated at pH 11
QPI: quinoa protein isolate.
